# Social activity as a mediator between childhood adversity and depressive symptoms in middle-aged and older Chinese adults

**DOI:** 10.3389/fpsyt.2025.1553895

**Published:** 2025-05-29

**Authors:** Ping Wu, Jinqi Ding, Haiying Jin, Junhang Zhang, Andrew J. Greenshaw, Sugai Liang

**Affiliations:** ^1^ Department of the Fourth School of Clinical Medicine, Zhejiang Chinese Medical University, Hangzhou, Zhejiang, China; ^2^ Huzhou Third Municipal Hospital, Affiliated Hospital of Huzhou University, Huzhou, Zhejiang, China; ^3^ Affiliated Mental Health Center & Hangzhou Seventh People’s Hospital, School of Medicine, Zhejiang University, Hangzhou, Zhejiang, China; ^4^ Department of Psychiatry, University of Alberta, Edmonton, AB, Canada

**Keywords:** adverse childhood experiences, social activity, depressive symptoms, middle-aged and older adults, Chinese

## Abstract

**Background:**

Adverse childhood experiences (ACEs) are associated with an increased risk of depressive symptoms (DS) in older adults. This study investigated the role of social activity in mediating the relationship between ACEs and DS among middle-aged and older Chinese adults.

**Methods:**

Data were derived from the China Health and Retirement Longitudinal Study (CHARLS). The discovery dataset included 10,164 participants from 2018, matched with life history data from 2014, while the replication dataset comprised 8,899 participants from 2020. DS was measured using the Center for Epidemiologic Studies Depression Scale (CES-D). Multiple linear regression and mediation analysis were conducted.

**Results:**

In the discovery dataset, ACEs were positively correlated with DS (r = 0.17, p < 0.001), while social activity was negatively correlated with both DS (r = –0.11, p < 0.001) and ACEs (r = –0.03, p = 0.01). Mediation analysis indicated that ACEs significantly predicted DS (estimate = 0.51, 95% CI 0.43 to 0.60), and social activity partially mediated this relationship (estimate = –0.01, bootstrap 95% CI –0.01 to –0.001), particularly among middle-aged adults (indirect effect estimate = –0.01, bootstrap 95% CI –0.01 to –0.001). Additionally, social activity notably mediated the relationship between childhood violence exposure and DS (estimate = –0.02, bootstrap 95% CI –0.04 to –0.003). These results were robustly validated through replication analysis, reinforcing the reliability of our conclusions.

**Conclusions:**

Social activity mediates the relationship between ACEs and DS, highlighting the importance of social engagement to reduce depression risk in this population.

## 1 Introduction

Late-life depression is a prevalent mental disorder that significantly impacts quality of life, leads to functional impairment (1), and increases suicide risk among older adults (2). Although the course of depression and depressive symptoms (DS) varies among individuals over a lifetime (3), DS are common in older adults (4) and are often overlooked by families and clinicians. The burden of DS negatively affects quality of life in this demographic (5). Identifying modifiable risk factors for DS is essential for improving public health through preventive clinical interventions.

Adverse childhood experiences (ACEs), including physical, emotional, and sexual abuse, neglect, and exploitation, significantly impair mental health and increase the risk of later-life depression (6). Early-life adversity may lower an individual’s stress threshold, increasing susceptibility to depressive responses to stressors (7). ACEs are also linked to increased disability (8) and psychological issues (9). Specific types of ACEs have varying relationships with DS. Childhood exposure to domestic violence predisposes individuals to psychiatric disorders such as depression, anxiety, and substance use (10). A family history of mental illness may indicate elevated genetic susceptibility to similar conditions (11). Parental mental health issues may impede children’s development and limit their engagement in educational and social activities (9). Additionally, ACEs are associated with social isolation, reduced social functioning in young adulthood (12), and diminished social participation in later life (9). Conversely, social participation significantly alleviates DS (9, 13), reduces depression risk, enhances life satisfaction, and improves mental health in older adults (14). Social interactions and support help older adults adapt to life changes and buffer stress (15). However, the potential for social activity to mediate the adverse effects of ACEs on DS remains unclear.

Using data from the China Health and Retirement Longitudinal Study (CHARLS), including follow-up surveys from 2018 and 2020, and the 2014 life history survey, this study investigated the impact of different ACEs categories on social activity and DS among middle-aged and older Chinese adults. The CHARLS cohort began in 2011 with biennial follow-ups. The present study aimed to assess whether social activity mediates the relationship between ACEs and DS and whether the impact of different ACEs categories varies in these population. We hypothesized that social activity would mediates the relationship between ACEs and DS and that the impact of different ACE categories could vary across individuals.

**Figure 1:**
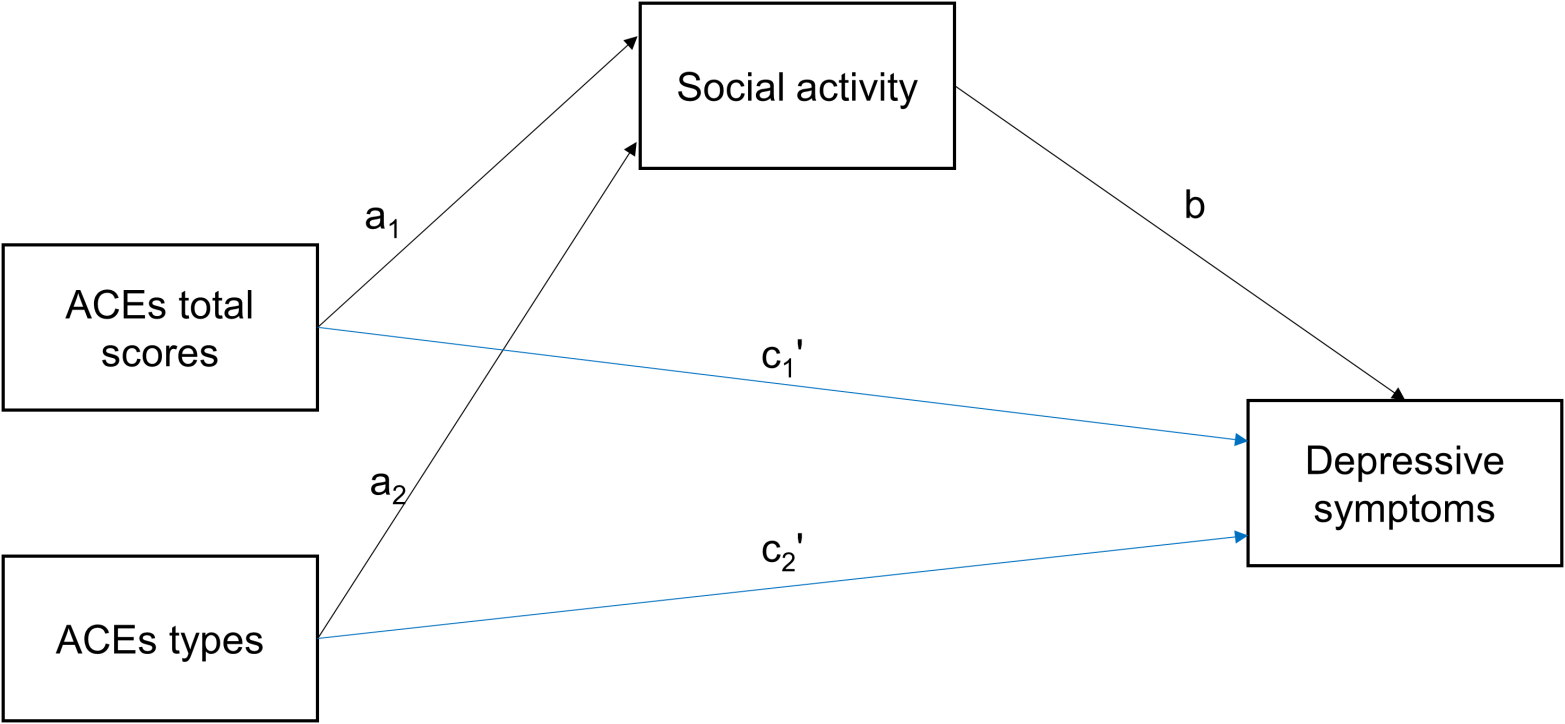
Mediation pathways. ACEs, adverse childhood experiences. Black arrows indicate indirect pathways, blue arrows indicate direct pathway. a_1_, a_2_, and b represent the indirect effects, while c_1_' and c_2_' represent the direct effects.

This study uniquely contributes to existing literature by employing a robust longitudinal design using two independent datasets, significantly enhancing the reliability and replicability of findings. Moreover, it explicitly clarifies the protective role of social activity as a mediator between ACEs and DS (**Figure 1**)—an aspect previously under-explored—particularly among middle-aged and older Chinese adults. These insights have important implications for preventive strategies and targeted interventions aimed at reducing depression risk in populations affected by early-life adversity.

## 2 Methods

### 2.1 Study design and participants

**Figure 2:**
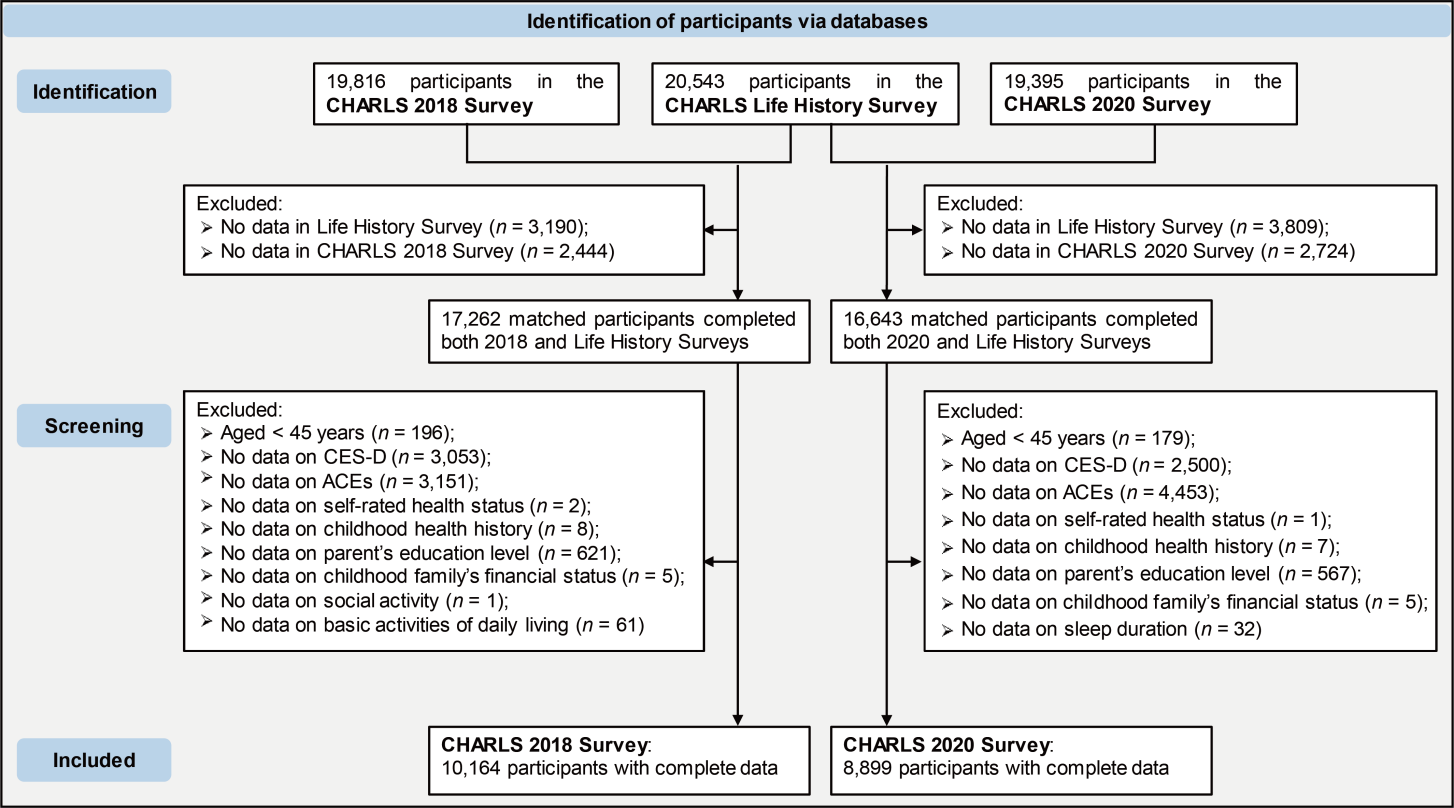
Data processing flowchart. CHARLS, the China Health and Retirement Longitudinal Study. ACEs, adverse childhood experiences. CES-D, the Center for Epidemiologic Studies Depression Scale.

This study utilized data from the CHARLS project, a comprehensive survey representing middle-aged and older populations across mainland China, with details on design and sampling previously reported (16–18). The analysis included data from the 2014 life history survey and measures of social activity and DS from 2018 and 2020, as well as demographic characteristics, socioeconomic status, health status, insurance, and healthcare use. Participants under 45 and those with missing data were excluded. A total of 10,164 participants from 2018, matched with 2014 life history data, were included as the discovery dataset, while 8,899 participants from 2020 were used as the replication dataset. Data processing steps are illustrated in **Figure 2**. Ethical approval was obtained from Peking University’s Ethics Review Board (IRB00001052-11015), and informed consent had been obtained from all participants as part of the CHARLS database project protocol. The research team received authorization to access to the CHARLS database.

### 2.2 Measures

#### 2.2.1 Depressive symptoms

DS were assessed using the Center for Epidemiologic Studies Depression Scale (CES-D), validated for use in middle-aged and older Chinese adults (19, 20). Higher scores indicated greater DS severity.

#### 2.2.2 Adverse childhood experiences

ACEs were assessed using the life history survey, which identified 12 ACEs indicators grouped into four categories: child maltreatment (physical abuse and emotional neglect), exposure to violence (domestic violence, peer bullying and unsafe neighborhood), parent/sibling death or disability (parental death, parental disability and sibling death), and parental maladjustment (household mental illness, substance abuse, parental separation or divorce and incarcerated household member). A higher cumulative score indicated greater ACEs exposure. Detailed ACEs metrics are provided in Supplementary Material: **Supplementary Table S1**.

#### 2.2.3 Social activity

Social activity was measured using specific items from the CHARLS follow-up questionnaire, encompassing participation in volunteer work, community groups, cultural events, and social gatherings (21). The social activity index was computed based on participation frequency over the past month (21, 22). See Supplementary Methods for further details.

### 2.3 Control variables

Control variables included demographic characteristics (age, male or female birth gender, education level, marital status), physical health (chronic diseases, self-rated health status, childhood health history, activities of daily living), lifestyle behaviors (smoking, drinking, nighttime sleep duration), socioeconomic status, and social security status. Socioeconomic status in adulthood was assessed based on education level, while childhood socioeconomic status was determined by parents’ education levels and family economic status during childhood. Social security measures included medical insurance and pension coverage. See Supplementary Methods for detailed descriptions.

### 2.4 Statistics analysis

All analyses were conducted using R version 4.2.2, with two-tailed tests and significance set at *p* < 0.05. Continuous data were presented as median [interquartile range], and categorical data as frequency and percentage. Associations between variables were assessed using Spearman’s correlation. Multiple linear regression models were employed to examine (1): the relationship between ACEs and social activity (Model 1) (2), the association between ACEs and DS (Model 2), and (3) the impact of ACEs on DS with social activity as a mediator (Model 3). Mediation analysis followed the Baron and Kenny approach (23) and was confirmed with PROCESS Model 4 (version 4.1) (24). Bootstrap resampling (5000 iterations) was used to generate 95% confidence intervals (CIs) to evaluate mediation effects. Significant mediation was indicated by non-zero CIs, and opposite signs of indirect and direct effects suggested a masking effect of the mediator. Subgroup analysis was performed based on age, categorizing individuals into middle-aged (< 65) and elderly (≥ 65) groups.

### 2.5 Validation analysis

Data from 2018 were used as the discovery dataset for initial findings, while data from 2020 served as the replication dataset for validation. To account for demographic differences between ACEs groups and controls, propensity score matching (25) was applied to the discovery dataset, followed by mediation analysis on the matched data. See Supplementary Methods for details.

## 3 Results

### 3.1 Participant characteristics

In both the discovery and replication datasets, the median CES-D scores were 7, the median ACEs scores were 1, and the median social activity indexes were 1. See **Supplementary Table S2** for detailed participant characteristics. In the discovery dataset, 4,389 participants (43.18%) experienced child maltreatment, 2,747 (27.03%) were exposed to violence, 4,551 (44.78%) faced parent/sibling death or disability, 1,343 (13.21%) had parental maladjustment, and 2,599 (25.57%) reported no ACEs. Significant differences were observed between ACEs categories in the CES-D scores (*p* < 0.001), social activity index (*p* = 0.001), and control variables, except for medical insurance and pension (**Table 1**). Participant characteristics of ACEs categories in the replication dataset are presented in **Supplementary Table S3**.

**Table 1:** Participant characteristics of ACEs categories in the discovery dataset.

Variable	Child maltreatment (*n* = 4,389)	Exposure to violence (*n* = 2,747)	Parent/sibling death or disability (*n* = 4,551)	Parental maladjustment (*n* = 1,343)	Controls (*n* = 2,599)	*p* value
CES-D scores						< 0.001^a^
Median [IQR]	7.0 [3.0, 12.0]	9.0 [4.0, 14.0]	8.0 [4.0, 13.0]	11.0 [5.0, 17.0]	6.0 [3.0, 10.0]	
Social activity index						0.001^a^
Median [IQR]	1.0 [0, 3.0]	1.0 [0, 3.0]	1.0 [0, 3.0]	1.0 [0, 3.0]	1.0 [0, 3.0]	
Age (years)						< 0.001^b^
< 65	2,970 (67.67%)	1,949 (70.95%)	2,756 (60.56%)	819 (60.98%)	1,821 (70.07%)	
≥ 65	1,419 (32.33%)	798 (29.05%)	1,795 (39.44%)	524 (39.02%)	778 (29.93%)	
Male or Female Birth Gender						< 0.001^b^
Female	1,975 (45.00%)	1,360 (49.51%)	2,274 (49.97%)	726 (54.06%)	1,426 (54.87%)	
Male	2,414 (55.00%)	1,387 (50.49%)	2,277 (50.03%)	617 (45.94%)	1,173 (45.13%)	
Education level						< 0.001^b^
< Middle school	2,585 (58.90%)	1,707 (62.14%)	3,013 (66.21%)	1,006 (74.91%)	1,420 (54.64%)	
≥ Middle school	1,804 (41.10%)	1,040 (37.86%)	1,538 (33.79%)	337 (25.09%)	1,179 (45.36%)	
Marital status						0.001^b^
No spouse	496 (11.30%)	317 (11.54%)	574 (12.61%)	194 (14.45%)	252 (9.70%)	
Have a spouse	3,893 (88.70%)	2,430 (88.46%)	3,977 (87.39%)	1,149 (85.55%)	2,347 (90.30%)	
Chronic diseases						< 0.001^b^
No	882 (20.10%)	500 (18.20%)	779 (17.12%)	205 (15.26%)	658 (25.32%)	
Yes	3,507 (79.90%)	2,247 (81.80%)	3,772 (82.88%)	1,138 (84.74%)	1,941 (74.68%)	
BADL disability						< 0.001^b^
No	3,744 (85.30%)	2,269 (82.60%)	3,717 (81.67%)	1,024 (76.25%)	2,264 (87.11%)	
Yes	645 (14.70%)	478 (17.40%)	834 (18.33%)	319 (23.75%)	335 (12.89%)	
IADL disability						< 0.001^b^
No	3,577 (81.50%)	2,140 (77.90%)	3,507 (77.06%)	931 (69.32%)	2,194 (84.42%)	
Yes	812 (18.50%)	607 (22.10%)	1,044 (22.94%)	412 (30.68%)	405 (15.58%)	
Self-rated health status						< 0.001^b^
Unhealthy	3,331 (75.89%)	2,143 (78.01%)	3,559 (78.20%)	1,072 (79.82%)	1,758 (67.64%)	
Healthy	1,058 (24.11%)	604 (21.99%)	992 (21.80%)	271 (20.18%)	841 (32.36%)	
Childhood health history						< 0.001^b^
Unhealthy	591 (13.47%)	477 (17.36%)	704 (15.47%)	263 (19.58%)	181 (6.96%)	
Healthy	3,798 (86.53%)	2,270 (82.64%)	3,847 (84.53%)	1,080 (80.42%)	2,418 (93.04%)	
Smoking						< 0.001^b^
No	3,011 (68.60%)	1,948 (70.91%)	3,249 (71.39%)	987 (73.49%)	1,943 (74.76%)	
Yes	1,378 (31.40%)	799 (29.09%)	1,302 (28.61%)	356 (26.51%)	656 (25.24%)	
Drinking						< 0.001^b^
No	2,628 (59.88%)	1,691 (61.56%)	2,932 (64.43%)	901 (67.09%)	1,707 (65.68%)	
Yes	1,761 (40.12%)	1,056 (38.44%)	1,619 (35.57%)	442 (32.91%)	892 (34.32%)	
Sleep duration						< 0.001^b^
Abnormal	1,713 (39.03%)	1,157 (42.12%)	1,964 (43.16%)	662 (49.29%)	901 (34.67%)	
Normal	2,676 (60.97%)	1,590 (57.88%)	2,587 (56.84%)	681 (50.71%)	1,698 (65.33%)	
Parent’s education level						< 0.001^b^
< Middle school	3,838 (87.45%)	2,395 (87.19%)	4,135 (90.86%)	1,207 (89.87%)	2,241 (86.23%)	
≥ Middle school	551 (12.55%)	352 (12.81%)	416 (9.14%)	136 (10.13%)	358 (13.77%)	
Childhood family’s financial status						< 0.001^b^
Worse than others	1,829 (41.67%)	1,377 (50.13%)	2,051 (45.07%)	777 (57.86%)	660 (25.39%)	
Equal to or better than others	2,560 (58.33%)	1,370 (49.87%)	2,500 (54.93%)	566 (42.14%)	1,939 (74.61%)	
Medical insurance						0.05^b^
No	122 (2.78%)	68 (2.48%)	113 (2.48%)	39 (2.90%)	44 (1.69%)	
Yes	4,267 (97.22%)	2,679 (97.52%)	4,438 (97.52%)	1,304 (97.10%)	2,555 (98.31%)	
Pension						0.28^b^
No	416 (9.48%)	286 (10.41%)	455 (10.00%)	128 (9.53%)	227 (8.73%)	
Yes	3,973 (90.52%)	2,461 (89.59%)	4,096 (90.00%)	1,215 (90.47%)	2,372 (91.27%)	

ACEs, adverse childhood experiences; CES-D, the Center for Epidemiologic Studies Depression Scale; BADL, basic activities of daily living; IADL, instrumental activities of daily living, CES-D scores and social activity index are presented as median [interquartile range]. Other variables are presented as frequency and percentage.

a Kruskal-Wallis rank sum test.

b Chi-squared test.

### 3.2 Correlation between ACEs scores, social activity index and CES-D scores

In the discovery dataset, CES-D scores were positively correlated with ACEs scores (*r* = 0.17, *p* < 0.001) and negatively correlated with the social activity index (*r* = –0.11, *p* < 0.001). ACEs scores were also negatively correlated with the social activity index (*r* = –0.03, *p* = 0.01).

### 3.3 Social activity as a mediator between ACEs and DS

Three multiple linear regression models are summarized in **Supplementary Table S4**, all adjusted for control variables. In the discovery dataset, Model 1 showed a significant association between ACEs scores and social activity index (B = 0.05, *p* = 0.01). Model 2 indicated a significant association between ACEs scores and CES-D scores (B = 0.51, *p* < 0.001). Model 3 demonstrated that the association between ACEs scores and CES-D scores remained significant (B = 0.51, *p* < 0.001) after adjusting for the social activity index, indicating partial mediation.

The bootstrap analysis confirmed a significant mediation effect of social activity on the relationship between ACEs and DS (indirect effect estimate = –0.01, bootstrap 95% CI –0.01 to –0.001), with a mediation effect of 1.96% (**Table 2**).

**Table 2:** Social activity as a mediator between ACEs categories and DS in the discovery dataset.

Independent variables	Model fit		Total effect		Direct effect		Indirect effect		Mediation proportion
	*R* ^2^	*F*	B (LLCI, ULCI)	SE	B (LLCI, ULCI)	SE	B (LLCI, ULCI)	SE	
ACEs	0.26	198.86^***^	0.51^***^ (0.42, 0.59)	0.04	0.51^***^ (0.43, 0.60)	0.04	–0.01 (–0.01, –0.001)	0.003	1.96%
Child maltreatment	0.25	130.44^***^	0.88^***^ (0.61, 1.16)	0.14	0.90^***^ (0.63, 1.17)	0.14	–0.02 (–0.03, –0.003)	0.01	2.27%
Exposure to violence	0.27	111.97^***^	1.54^***^ (1.22, 1.85)	0.16	1.55^***^ (1.24, 1.87)	0.16	–0.02 (–0.04, –0.003)	0.01	1.30%
Parent/sibling death or disability	0.26	142.02^***^	0.87^***^ (0.58, 1.15)	0.14	0.88^***^ (0.60, 1.16)	0.14	–0.01 (–0.03, 0.004)	0.01	NA
Parental maladjustment	0.30	94.87^***^	2.34^***^ (1.93, 2.75)	0.21	2.36^***^ (1.95, 2.77)	0.21	–0.02 (–0.05, 0.0002)	0.01	NA

Estimated by the bias-corrected percentile bootstrap method.

ACEs, adverse childhood experiences; DS, depressive symptoms; B, effect estimate coefficient; LLCI, lower limits of 95% confidence interval; ULCI, upper limits of 95% confidence interval; SE, standard error. Mediation proportion = | Indirect effect |/Total effect. NA indicates that the mediation effect for the corresponding path was not significant.

***p < 0.001.

### 3.4 Mediating role of social activity in ACEs categories and DS

In the discovery dataset, social activity partially mediates the relationship between exposure to violence (indirect effect estimate = –0.02, bootstrap 95% CI –0.04 to –0.003) and child maltreatment (indirect effect estimate = –0.02, bootstrap 95% CI –0.03 to –0.003) on DS. See **Table 2**. Parental maladjustment had the largest effect on DS (total effect estimate = 2.34, 95% CI 1.93 to 2.75; direct effect estimate = 2.36, 95% CI 1.95 to 2.77). However, social activity did not mediate the effects of parental maladjustment or parent/sibling death or disability on DS.

### 3.5 Subgroup analysis

We divided middle-aged and elderly into two groups and conducted an analysis to explore the mediating effects separately. The results showed that the mediating effect of social activity differed from that of age (**Supplementary Table S5**). In the discovery dataset, for the middle-aged group, social activity partially mediates the relationship between ACEs and DS (indirect effect estimate = –0.01, bootstrap 95% CI –0.01 to –0.001). We observed analogous mediating effects of social activity among the middle-aged group concerning the impact of exposure to violence and parental maladjustment on DS. However, the mediating effect in the elderly group was not significant.

### 3.6 Validation analysis

Post-matching balance testing showed no significant differences in age (*p* = 0.93) or gender (*p* = 0.99) between ACEs categories and controls in the discovery dataset (**Supplementary Table S6**). Participant characteristics after matching are listed in **Supplementary Table S7**. Mediation analysis results in the post-matching dataset were consistent with the main findings (**Supplementary Table S8**).

In the replication dataset, CES-D scores were positively correlated with ACEs scores (*r* = 0.17, *p* < 0.001) and negatively correlated with the social activity index (*r* = –0.08, *p* < 0.001). However, the correlation between ACEs scores and the social activity index was not statistically significant (*r* = –0.004, *p* = 0.72). Multiple linear regression results were consistent with those in the discovery dataset (see **Supplementary Table S9**). Social activity mediated the relationship between ACEs and DS, with significant effects observed for exposure to violence (indirect effect estimate = –0.03, bootstrap 95% CI –0.05 to –0.01) and parental maladjustment (indirect effect estimate = –0.02, bootstrap 95% CI –0.05 to –0.002), as shown in **Supplementary Table S10**.

## 4 Discussion

Using data from a large-scale, population-based cohort with longitudinal follow-up, this study investigated the relationships between ACEs, social activity, and DS in middle-aged and older adults in China. ACEs were associated with reduced social participation and increased DS in later life. Notably, social participation in later life may mediates the relationship between ACEs and DS, particularly for individuals exposed to childhood violence. Among ACEs categories, parental maladjustment had the most significant impact on DS.

Mounting evidence supports a significant association between ACEs and depression (17, 26). ACEs can lead to negative self-perception, maladaptive stress responses, and diminished self-worth (27), thereby increasing the risk of DS. Consistent with previous research (17), this study found that DS severity was positively correlated with ACEs scores and negatively correlated with the social activity index. Participation in social activities serves as a protective factor against DS, particularly for those who experienced childhood violence. Social activities, such as peer-led support groups and social skills training (13), foster relationships and emotional connections, thereby improving mental well-being (9). Social participation enhances personal relationships and provides emotional support (28), alleviating loneliness (29) and consequently reducing DS. Participation in various social activities helps older adults build a group identity, thereby enhancing self-esteem and self-acceptance (30). Furthermore, social activity may improve cognitive reserve (31), facilitating the re-evaluation and interpretation of life events, and thus alleviating depression (32).

In line with prior research (17, 33), the current findings indicate that exposure to childhood violence, including domestic violence, unsafe neighborhoods, and peer bullying, is linked to increased DS among middle-aged and older adults. Such exposure may have persistent biological effects on stress response systems, resulting in long-lasting physical and psychological vulnerabilities (34). Peer bullying, in particular, may impair social skills, leading to antisocial behaviors (e.g., aggression, substance abuse, school dropout) (35) and resulting in social isolation. Childhood violence exposure can also impair interpersonal relationships, increasing mistrust and reducing social interaction (36). Older adults who experienced childhood violence often receive limited support from social networks and perceive lower levels of social support (30). These challenges are particularly pronounced for individuals whose social skills and networks were disrupted by early exposure to violence (37). Engaging in social activities may help these individuals rebuild connections and foster resilience against the lingering effects of early-life violence.

This study found that parental maladjustment had the most substantial impact on DS, consistent with previous studies (17). Poor parental mental health and adverse family environments significantly affect child development (11, 38). Twin and family studies have consistently demonstrated a genetic predisposition to mental disorders, with notable familial clustering (11, 39, 40). Parental mental health issues and adverse family environments may influence children’s brain development, increasing susceptibility to stress and depression later in life (40, 41). However, social participation did not statistically significantly mediate the relationship between parental maladjustment and DS, suggesting that genetic factors may play a more substantial role. The present study also found that the mediating effect differed between age subgroups. This is likely due to somatic factors exerting a stronger influence on DS in elderly adults compared to psychosocial factors (42).

This study has several limitations. First, reliance on self-reported, retrospective accounts of childhood experiences may introduce recall bias (43). Second, the current study employs a cumulative ACEs score, which does not differentiate between isolated adverse events and prolonged or severe childhood adversity. Future research should incorporate weighted ACE scoring systems that account for intensity and chronicity to provide a more nuanced understanding of long-term psychological impacts (44). Third, the analysis based on the CHARLS database lacked qualitative insights and individual behavioral data. Fourth, middle-aged and older adults with low levels of social activity are overrepresented due to the restricted distribution of the social activity index, potentially attenuating the observed mediating effect between ACEs and depressive symptoms. The index captures both the number and frequency of social activities, but a one-point increase may indicate either a slight behavioral change or the addition of another infrequent activity. Therefore, findings should be interpreted cautiously, and future research is encouraged to refine this measure and validate its predictive value in longitudinal settings.

## 5 Conclusions

This longitudinal study revealed significant associations between ACEs, social activity, and DS. Social activity may serve as a protective factor in the relationship between ACEs and DS, underscoring the importance of ACE prevention and comprehensive public health strategies. Interventions promoting social engagement for individuals with ACEs may mitigate the impact of depressive symptoms in middle-age and older adults.

## Data Availability

Publicly available datasets were analyzed in this study. This data can be found here: Data were gathered from the China Health and Retirement Longitudinal Study (CHARLS), a publicly available database that could be accessed at http://charls.pku.edu.cn.
