# Supplementary material for: Social activity as a mediator between childhood adversity and depressive symptoms in middle-aged and older Chinese adults
Source: Front Psychiatry. 2025 May 29;16:1553895. doi: 10.3389/fpsyt.2025.1553895 (PMC12160938; doi:10.3389/fpsyt.2025.1553895)
Supplement: Supplementary file 1 [file Table1.docx]

**Supplementary Material for *Social activity as a mediator between childhood adversity and depressive symptoms in middle-aged and older Chinese adults***

**Supplementary Methods**

Calculation of the social activity index

Selection of control variables

Propensity score matching procedure

**Supplementary Tables**

Table S1 Details of specific measures for each ACEs indicator item (refer (4, 6))

Table S2 Participant characteristics in the discovery and replication datasets

Table S3 Participant characteristics of ACEs categories in the replication dataset

Table S4 Multiple linear regression analysis in the discovery dataset

Table S5 Mediation effects of social activity between ACEs and DS stratified by age

Table S6 Balance test for post-matching discovery dataset

Table S7 Participant characteristics of ACEs categories in the post-matching discovery dataset

Table S8 Social activity as a mediator between ACEs categories and DS in the post-matching discovery dataset

Table S9 Multiple linear regression analysis in the replication dataset

Table S10 Social activity as a mediator between ACEs categories and DS in the replication dataset

**Supplementary Methods**

**Calculation of the social activity index**

Social activity was assessed and calculated based on the questions in the CHARLS: 'Have you engaged in any of these activities in the past month?' and 'How frequently did you engage in these activities over the past month: almost daily, almost every week, or not regularly?' In the 2018 CHARLS questionnaires, there were 11 social activities assessed: (1) interacting with friends, (2) playing Ma-jong, chess, cards, or going to a community club, (3) providing help to family, friends, or neighbors who do not live with you, (4) going to a sport, social, or other kind of club, (5) taking part in a community-related organization, (6) doing voluntary or charity work, (7) caring for a sick or disabled adult who does not live with you, (8) attending an educational or training course, (9) stock investment, (10) using the Internet, and (11) other activities. In the 2020 CHARLS questionnaires, there were 8 social activities assessed: (1) interacting with friends, (2) playing Ma-jong, chess, cards, or going to a community club, (3) providing help to family, friends, or neighbors who do not live with you, (4) going to a sport, social, or other kind of club, (5) taking part in a community-related organization, (6) doing voluntary or charity work, caring for a sick or disabled adult who does not live with you, (7) attending an educational or training course, and (8) other social activities.

According to previous studies (1-3), the social activity index was calculated based on the social activities listed in the questionnaire and their frequencies. In the formula, **social activity index**$=\sum_{i=1}^{n=11} \left( A_{i}*F_{i} \right)$

$A_{i}$indicates whether an individual engaged in social event (Yes = 1, No = 0), and $F_{i}$ denotes frequency of participation in each activity (almost daily = 3, almost every week = 2, not regularly = 1).

**Selection of control variables**

The selection of control variables was guided by previous research (4) using data from the CHARLS questionnaire, including demographic characteristics, physical health, lifestyle behaviors, socioeconomic status, and social security indicators.

*Demographic characteristics* included age (0 = under 65 years, 1 = 65 years or older), male or female birth gender (0 = female, 1 = male), education level (0 = below middle school, 1 = middle school or above), and marital status (0 = separated / divorced / widowed/never married, 1 = married/cohabiting).

*Physical health* *indicators* included self-reported presence of chronic diseases (0 = no, 1 = yes), self-rated health status (0 = fair/poor/very poor, 1 = very good/good), childhood health history (0 = somewhat less healthy/much less healthy, 1 = about average/somewhat healthier/much healthier), basic activities of daily living (BADL), and instrumental activities of daily living (IADL). BADL indicators included dressing, bathing, eating, getting in and out of bed, using the toilet, and maintaining continence. A normal BADL score was defined as reporting 'no difficulty' in all six areas; any other response indicated a BADL disability. Normal IADL was defined as reporting 'no difficulty' in five areas: doing housework, cooking, shopping, taking medication, and managing money. Any response other than 'no difficulty' indicated an IADL disability.

*Lifestyle behaviors* included smoking (0 = no, 1 = yes), drinking (0 = no, 1 = yes), and nighttime sleep duration (0 = abnormal, 1 = normal). Abnormal sleep duration was defined as either too short (≤ 5 hours per day) or too long (≥ 9 hours per day).

*Adult socioeconomic status* was assessed based on the respondent's education level.

*Childhood socioeconomic status* was evaluated using two criteria: the education level of the respondents' parents during their childhood (0 = below middle school, 1 = middle school or above) and childhood family financial status (0 = worse than others, 1 = equal to or better than others).

*Social security indicators* included the presence of medical insurance (0 = no, 1 = yes) and a retirement pension (0 = no, 1 = yes).

**Propensity score matching procedure**

Propensity score matching was performed using the R package *MatchIt* (5). To retain a high percentage of data, matching was not conducted at a 1:1 ratio due to the unequal group sizes in the discovery dataset: 4,389 participants in the child maltreatment group, 2,747 in the exposure to violence group, 4,551 in the parent/sibling death or disability group, 1,343 in the parental maladjustment group, and 2,599 in the control group. Specifically, the parental maladjustment group was matched to the control group at a 1:1 ratio, while the control group was matched to the child maltreatment, exposure to violence, and parent/sibling death or disability groups at ratios of 1:2, 1:1, and 1:3, respectively. These matching ratios were determined using a nearest neighbor matching algorithm with a caliper of 0.05. The Kruskal-Wallis rank sum test for age and the chi-square test for male or female birth gender were used to compare the ACEs categories and controls both pre- and post-matching in the discovery dataset.

**Table S1** Details of specific measures for each ACEs indicator item (refer (4, 6))

| ACEs categories | Indicator^▲^ | Measure (item) | Response |
| --- | --- | --- | --- |
| Child maltreatment | Physical abuse | When you were growing up, did your female/male guardian ever hit you? | Often^★^, sometimes^★^, rarely or never |
|  | Emotional neglect | How much love and affection did your female guardian give you while you were growing up? | Often, sometimes, rarely^★^ or never^★^ |
| Exposure to violence | Domestic violence | Have your father/mother ever beat up your mother/father? | Often^★^, sometimes^★^, not very often, or never |
|  | Peer bullying | When you were a child, how often were you picked on or bullied by kids in your neighborhood/ school? | Often^★^, sometimes^★^, not very often, or never |
|  | Unsafe neighborhood | Was it safe being out alone at night in the neighborhood where you lived as a child? | Very safe, somewhat safe, not very safe^★^, or not safe at all^★^ |
| Parent/sibling death or disability | Parental death^◆^ | Either of the parents was dead before you were 17 years? | (Yes^★^ or No) |
|  | Parental disability | Did your female/male guardian have a long time being sick on bed when you were young? | (Yes^★^ or No) |
|  |  | Did your female/male guardian have a serious deformity when you were young? | (Yes^★^ or No) |
|  | Sibling death^◆^ | Any of the siblings was dead before you were 17 years? | (Yes^★^ or No) |
| Parental maladjustment | Household mental illness | Did your female/male guardian have abnormality of mind when you were young? | (Yes^★^ or No) |
|  |  | During the years you were growing up, had your female/male guardian often showed continued signs of sadness or depression? | (During all^★^ , most^★^ , some, or only a little of the childhood) |
|  | Substance abuse | During the years you were growing up, did your female/male guardian ever have alcoholism or drug? | (Yes^★^ or No) |
|  | Parental separation or divorce | Were your biological parents divorced (including long separation due to emotional problems) before you were 17 years? | (Yes^★^ or No) |
|  | Incarcerated household member | During the years you were growing up, have your female/male guardian ever been arrested or sent to prison? | (Yes^★^ or No) |

ACEs, adverse childhood experiences.

^▲^ Each ACE indicator was binary-coded (0 or 1) and summed to create a cumulative score for each participant (0-12). ^★^ threshold for each item.

^◆^ indicated that calculation based on the dates of birth and their parental or siblings’ death.

**Table S2** Participant characteristics in the discovery and replication datasets

| Variable | Discovery dataset  (*N* = 10,164) | Replication dataset  (*N* = 8,899) | Variable | Discovery dataset  (*N* = 10,164) | Replication dataset  (*N* = 8,899) |
| --- | --- | --- | --- | --- | --- |
| CES-D scores |  |  | Smoking |  |  |
| Median [IQR] | 7.0 [3.0, 12.0] | 7.0 [3.0, 12.0] | No | 7,298 (71.80%) | 6,331 (71.14%) |
| ACEs scores |  |  | Yes | 2,866 (28.20%) | 2,568 (28.86%) |
| Median [IQR] | 1.0 [0, 2.0] | 1.0 [1.0, 2.0] | Drinking |  |  |
| Social activity index |  |  | No | 6,446 (63.42%) | 5,180 (58.21%) |
| Median [IQR] | 1.0 [0, 2.0] | 1.0 [0, 3.0] | Yes | 3,718 (36.58%) | 3,719 (41.79%) |
| Age (years) |  |  | Sleep duration |  |  |
| < 65 | 6,774 (66.65%) | 6,202 (69.69%) | Abnormal | 3,956 (38.92%) | 3,390 (38.09%) |
| ≥ 65 | 3,390 (33.35%) | 2,697 (30.31%) | Normal | 6,208 (61.08%) | 5,509 (61.91%) |
| Male or Female Birth Gender |  |  | Parent’s education level |  |  |
| Female | 5,118 (50.35%) | 4,122 (46.32%) | < Middle school | 8,939 (87.95%) | 7,741 (86.99%) |
| Male | 5,046 (49.65%) | 4,777 (53.68%) | ≥ Middle school | 1,225 (12.05%) | 1,158 (13.01%) |
| Education level |  |  | Childhood family’s financial status |  |  |
| < Middle school | 6,090 (59.92%) | 4,876 (54.79%) | Worse than others | 3,815 (37.53%) | 3,278 (36.84%) |
| ≥ Middle school | 4,074 (40.08%) | 4,023 (45.21%) | Equal to or better than others | 6,349 (62.47%) | 5,621 (63.16%) |
| Marital status |  |  | Medical Insurance |  |  |
| No spouse | 1,147 (11.28%) | 1,030 (11.57%) | No | 233 (2.29%) | 293 (3.29%) |
| Have a spouse | 9,017 (88.72%) | 7,869 (88.43%) | Yes | 9,931 (97.71%) | 8,606 (96.71%) |
| Chronic diseases |  |  | Pension |  |  |
| No | 2,113 (20.79%) | 1,766 (19.84%) | No | 968 (9.52%) | 1,218 (13.69%) |
| Yes | 8,051 (79.21%) | 7,133 (80.16%) | Yes | 9,196 (90.48%) | 7,681 (86.31%) |
| BADL disability |  |  | Child maltreatment |  |  |
| No | 8,625 (84.86%) | 7,306 (82.10%) | No | 5,775 (56.82%) | 5,075 (57.03%) |
| Yes | 1,539 (15.14%) | 1,593 (17.90%) | Yes | 4,389 (43.18%) | 3,824 (42.97%) |
| IADL disability |  |  | Exposure to violence |  |  |
| No | 8,223 (80.90%) | 7,410 (83.27%) | No | 7,417 (72.97%) | 6,341 (71.26%) |
| Yes | 1,941 (19.10%) | 1,489 (16.73%) | Yes | 2,747 (27.03%) | 2,558 (28.74%) |
| Self-rated health status |  |  | Parent/sibling death or disability |  |  |
| Unhealthy | 7,517 (73.96%) | 6,639 (74.60%) | No | 5,613 (55.22%) | 4,828 (54.25%) |
| Healthy | 2,647 (26.04%) | 2,260 (25.40%) | Yes | 4,551 (44.78%) | 4,071 (45.75%) |
| Childhood health history |  |  | Parental maladjustment |  |  |
| Unhealthy | 1,218 (11.98%) | 1,100 (12.36%) | No | 8,821 (86.79%) | 7,762 (87.22%) |
| Healthy | 8,946 (88.02%) | 7,799 (87.64%) | Yes | 1,343 (13.21%) | 1,137 (12.78%) |

ACEs, adverse childhood experiences. CES-D, the Center for Epidemiologic Studies Depression Scale. BADL, basic activities of daily living. IADL, instrumental activities of daily living. CES-D scores and social activity index are presented as median [interquartile range]. Other variables are shown as frequency and percentage.

**Table S3** Participant characteristics of ACEs categories in the replication dataset

| Variable | Child maltreatment  (*n* = 3,824) | Exposure to violence  (*n* = 2,558) | Parent/sibling death or disability  (*n* = 4,071) | Parental maladjustment  (*n* = 1,137) | Controls  (*n* = 2,179) | *p* value |
| --- | --- | --- | --- | --- | --- | --- |
| CES-D scores |  |  |  |  |  | < 0.001^a^ |
| Median [IQR] | 7.0 [3.0, 12.0] | 8.0 [4.0, 14.0] | 8.0 [4.0, 13.0] | 10.0 [6.0, 16.0] | 6.0 [3.0, 10.0] |  |
| Social activity index |  |  |  |  |  | 0.25^a^ |
| Median [IQR] | 1.0 [0, 3.0] | 1.0 [0, 3.0] | 1.0 [0, 3.0] | 1.0 [0, 3.0] | 1.0 [0, 3.0] |  |
| Age (years) |  |  |  |  |  | < 0.001^b^ |
| < 65 | 2,736 (71.55%) | 1,894 (74.04%) | 2,601 (63.89%) | 756 (66.49%) | 1,592 (73.06%) |  |
| ≥ 65 | 1,088 (28.45%) | 664 (25.96%) | 1,470 (36.11%) | 381 (33.51%) | 587 (26.94%) |  |
| Male or Female Birth Gender |  |  |  |  |  | < 0.001^b^ |
| Female | 1,577 (41.24%) | 1,196 (46.76%) | 1,854 (45.54%) | 559 (49.16%) | 1,077 (49.43%) |  |
| Male | 2,247 (58.76%) | 1,362 (53.24%) | 2,217 (54.46%) | 578 (50.84%) | 1,102 (50.57%) |  |
| Education level |  |  |  |  |  | < 0.001^b^ |
| < Middle school | 2,058 (53.82%) | 1,509 (58.99%) | 2,489 (61.14%) | 789 (69.39%) | 1,047 (48.05%) |  |
| ≥ Middle school | 1,766 (46.18%) | 1,049 (41.01%) | 1,582 (38.86%) | 348 (30.61%) | 1,132 (51.95%) |  |
| Marital status |  |  |  |  |  | < 0.001^b^ |
| No spouse | 413 (10.80%) | 297 (11.61%) | 533 (13.09%) | 172 (15.13%) | 228 (10.46%) |  |
| Have a spouse | 3,411 (89.20%) | 2,261 (88.39%) | 3,538 (86.91%) | 965 (84.87%) | 1,951 (89.54%) |  |
| Chronic diseases |  |  |  |  |  | < 0.001^b^ |
| No | 726 (18.99%) | 420 (16.42%) | 681 (16.73%) | 174 (15.30%) | 541 (24.83%) |  |
| Yes | 3,098 (81.01%) | 2,138 (83.58%) | 3,390 (83.27%) | 963 (84.70%) | 1,638 (75.17%) |  |
| BADL disability |  |  |  |  |  | < 0.001^b^ |
| No | 3,083 (80.62%) | 1,973 (77.13%) | 3,192 (78.41%) | 813 (71.50%) | 1,908 (87.56%) |  |
| Yes | 741 (19.38%) | 585 (22.87%) | 879 (21.59%) | 324 (28.50%) | 271 (12.44%) |  |
| IADL disability |  |  |  |  |  | < 0.001^b^ |
| No | 3,172 (82.95%) | 1,996 (78.03%) | 3,219 (79.07%) | 831 (73.09%) | 1,912 (87.75%) |  |
| Yes | 652 (17.05%) | 562 (21.97%) | 852 (20.93%) | 306 (26.91%) | 267 (12.25%) |  |
| Self-rated health status |  |  |  |  |  | < 0.001^b^ |
| Unhealthy | 2,941 (76.91%) | 2,009 (78.54%) | 3,149 (77.35%) | 907 (79.77%) | 1,518 (69.66%) |  |
| Healthy | 883 (23.09%) | 549 (21.46%) | 922 (22.65%) | 230 (20.23%) | 661 (30.34%) |  |
| Childhood health history |  |  |  |  |  | < 0.001^b^ |
| Unhealthy | 521 (13.62%) | 464 (18.14%) | 629 (15.45%) | 233 (20.49%) | 156 (7.16%) |  |
| Healthy | 3,303 (86.38%) | 2,094 (81.86%) | 3,442 (84.55%) | 904 (79.51%) | 2,023 (92.84%) |  |
| Smoking |  |  |  |  |  | 0.007^b^ |
| No | 2,613 (68.33%) | 1,800 (70.37%) | 2,869 (70.47%) | 819 (72.03%) | 1,601 (73.47%) |  |
| Yes | 1,211 (31.67%) | 758 (29.63%) | 1,202 (29.53%) | 318 (27.97%) | 578 (26.53%) |  |
| Drinking |  |  |  |  |  | 0.001^b^ |
| No | 2,095 (54.79%) | 1,473 (57.58%) | 2,422 (59.49%) | 676 (59.45%) | 1,307 (59.98%) |  |
| Yes | 1,729 (45.21%) | 1,085 (42.42%) | 1,649 (40.51%) | 461 (40.55%) | 872 (40.02%) |  |
| Sleep duration |  |  |  |  |  | < 0.001^b^ |
| Abnormal | 1,475 (38.57%) | 1,059 (41.40%) | 1,700 (41.76%) | 513 (45.12%) | 734 (33.69%) |  |
| Normal | 2,349 (61.43%) | 1,499 (58.60%) | 2,371 (58.24%) | 624 (54.88%) | 1,445 (66.31%) |  |
| Parent’s education level |  |  |  |  |  | < 0.001^b^ |
| < Middle school | 3,289 (86.01%) | 2,205 (86.20%) | 3,657 (89.83%) | 991 (87.16%) | 1,868 (85.73%) |  |
| ≥ Middle school | 535 (13.99%) | 353 (13.80%) | 414 (10.17%) | 146 (12.84%) | 311 (14.27%) |  |
| Childhood family’s financial status |  |  |  |  |  | < 0.001^b^ |
| Worse than others | 1,565 (40.93%) | 1,281 (50.08%) | 1,798 (44.17%) | 654 (57.52%) | 526 (24.14%) |  |
| Equal to or better than others | 2,259 (59.07%) | 1,277 (49.92%) | 2,273 (55.83%) | 483 (42.48%) | 1,653 (75.86%) |  |
| Medical insurance |  |  |  |  |  | 0.49^b^ |
| No | 113 (2.96%) | 92 (3.60%) | 135 (3.32%) | 44 (3.87%) | 76 (3.49%) |  |
| Yes | 3,711 (97.04%) | 2,466 (96.40%) | 3,936 (96.68%) | 1,093 (96.13%) | 2,103 (96.51%) |  |
| Pension |  |  |  |  |  | 0.74^b^ |
| No | 535 (13.99%) | 329 (12.86%) | 541 (13.29%) | 156 (13.72%) | 299 (13.72%) |  |
| Yes | 3,289 (86.01%) | 2,229 (87.14%) | 3,530 (86.71%) | 981 (86.28%) | 1,880 (86.28%) |  |

ACEs, adverse childhood experiences. CES-D, the Center for Epidemiologic Studies Depression Scale. BADL, basic activities of daily living. IADL, instrumental activities of daily living. CES-D scores and social activity index are presented as median [interquartile range]. Other variables are presented as frequency and percentage.

^a^ Kruskal-Wallis rank sum test; ^b^ Chi-squared test.

**Table S4** Multiple linear regression analysis in the discovery dataset

|  | Social activity index | | CES-D scores | | CES-D scores | |
| --- | --- | --- | --- | --- | --- | --- |
|  | Model 1 | | Model 2 | | Model 3 | |
|  | B | SE | B | SE | B | SE |
| Age | –0.19^***^ | 0.06 | –1.24^***^ | 0.13 | –1.27^***^ | 0.13 |
| Male or Female Birth Gender | –0.50^***^ | 0.06 | –1.30^***^ | 0.14 | –1.37^***^ | 0.14 |
| Education level | 1.08^***^ | 0.05 | –0.94^***^ | 0.12 | –0.79^***^ | 0.12 |
| Marital status | –0.06^***^ | 0.08 | –1.35^***^ | 0.18 | –1.36^***^ | 0.18 |
| Chronic diseases | 0.31 | 0.06 | 0.85^***^ | 0.14 | 0.89^***^ | 0.14 |
| BADL disability | –0.14^***^ | 0.08 | 2.82^***^ | 0.18 | 2.80^***^ | 0.18 |
| IADL disability | –0.32 | 0.07 | 2.75^***^ | 0.16 | 2.71^***^ | 0.16 |
| Self-rated health status | 0.39^***^ | 0.06 | –2.30^***^ | 0.13 | –2.25^***^ | 0.13 |
| Childhood health history | –0.11 | 0.08 | –0.73^***^ | 0.17 | –0.74^***^ | 0.17 |
| Smoking | –0.01 | 0.06 | 0.68^***^ | 0.15 | 0.68^***^ | 0.15 |
| Drinking | 0.63^***^ | 0.06 | –0.49^***^ | 0.13 | –0.41^**^ | 0.13 |
| Sleep duration | 0.16^**^ | 0.05 | –1.91^***^ | 0.12 | –1.88^***^ | 0.12 |
| Parent’s education level | 0.62^***^ | 0.08 | 0.11 | 0.17 | 0.19 | 0.17 |
| Childhood family’s financial status | 0.23^***^ | 0.05 | –0.53^***^ | 0.12 | –0.50^***^ | 0.12 |
| Medical insurance | 0.15 | 0.17 | 0.29 | 0.38 | 0.31 | 0.38 |
| Pension | 0.12 | 0.08 | 0.10 | 0.19 | 0.11 | 0.19 |
| ACEs scores | 0.05^*^ | 0.02 | 0.51^***^ | 0.04 | 0.51^***^ | 0.04 |
| Social activity index |  |  |  |  | –0.13^***^ | 0.02 |
|  | *R*^2^ = 0.10 | | *R*^2^ = 0.26 | | *R*^2^ = 0.26 | |
|  | *F*_(17, 10146)_ = 65.6^***^ | | *F*_(17，10146)_ = 207.9^***^ | | *F*_(18, 10145)_ = 198.9^***^ | |

ACEs, adverse childhood experiences. CES-D, the Center for Epidemiologic Studies Depression Scale. BADL, basic activities of daily living. IADL, instrumental activities of daily living.

Model 1 examined the link between ACEs and social activity. Model 2 explored the relationship between ACEs and DS. Model 3 assessed whether social activity mediated the relationship between ACEs and DS. B, regression coefficient. SE, standard error.

^*^ *p* < 0.05, ^**^ *p* < 0.01, ^***^ *p* < 0.001.

**Table S5** Mediation effects of social activity between ACEs and DS stratified by age

| Dataset | Independent variables | Age  (years) | Model fit | | Total effect | | Direct effect | | Indirect effect | | Mediation proportion |
| --- | --- | --- | --- | --- | --- | --- | --- | --- | --- | --- | --- |
|  |  |  | *R*^2^ | *F* | B (LLCI, ULCI) | SE | B (LLCI, ULCI) | SE | B (LLCI, ULCI) | SE |  |
| Discovery | ACEs | <65 | 0.26 | 143.12^***^ | 0.50^***^ (0.40, 0.61) | 0.05 | 0.51^***^ (0.41, 0.61) | 0.05 | –0.01 (–0.01, –0.001) | 0.003 | 1.01% |
|  |  | ≥65 | 0.26 | 70.92^***^ | 0.50^***^ (0.35, 0.65) | 0.08 | 0.51^***^ (0.36, 0.66) | 0.08 | –0.01 (–0.02, 0.01) | 0.01 | NA |
|  | Child maltreatment | <65 | 0.26 | 98.23^***^ | 0.97^***^ (0.65, 1.30) | 0.16 | 0.98^***^ (0.66, 1.31) | 0.16 | –0.01 (–0.03, 0.003) | 0.01 | NA |
|  |  | ≥65 | 0.25 | 42.69^***^ | 0.68^**^ (0.17, 1.20) | 0.26 | 0.71^**^ (0.20, 1.22) | 0.26 | –0.03 (–0.08, 0.02) | 0.02 | NA |
|  | Exposure to violence | <65 | 0.27 | 81.37^***^ | 1.56^***^ (1.20, 1.93) | 0.19 | 1.58^***^ (1.21, 1.94) | 0.19 | –0.01 (–0.04, 0.001) | 0.01 | NA |
|  |  | ≥65 | 0.30 | 38.49^***^ | 1.47^***^ (0.87, 2.07) | 0.31 | 1.47^***^ (0.88, 2.07) | 0.31 | –0.001(–0.05, 0.05) | 0.03 | NA |
|  | Parent/sibling death or disability | <65 | 0.26 | 101.21^***^ | 0.98^***^ (0.63, 1.32) | 0.17 | 0.98^***^ (0.64, 1.32) | 0.17 | –0.00 (–0.02, 0.01) | 0.01 | NA |
|  |  | ≥65 | 0.28 | 57.25^***^ | 0.65^***^ (0.15, 1.14) | 0.25 | 0.69^***^ (0.20, 1.19) | 0.25 | –0.05 (–0.11, 0.01) | 0.03 | NA |
|  | Parental maladjustment | <65 | 0.30 | 67.06^***^ | 2.19^***^ (1.69, 2.69) | 0.26 | 2.19^***^ (1.69, 2.69) | 0.26 | –0.02 (–0.06, 0.001) | 0.02 | NA |
|  |  | ≥65 | 0.32 | 34.79^***^ | 2.60^***^ (1.90, 3.31) | 0.36 | 2.60^***^ (1.89, 3.30) | 0.36 | 0.01 (–0.07, 0.08) | 0.04 | NA |
| Replication | ACEs | <65 | 0.29 | 146.41^***^ | 0.44^***^ (0.34, 0.54) | 0.05 | 0.45^***^ (0.35, 0.55) | 0.06 | –0.01 (–0.01, –0.002) | 0.003 | 1.60% |
|  |  | ≥65 | 0.27 | 57.91^***^ | 0.44^***^ (0.28, 0.60) | 0.08 | 0.44 ^***^(0.28, 0.60) | 0.08 | –0.001 (–0.01, 0.01) | 0.01 | NA |
|  | Child maltreatment | <65 | 0.27 | 94.99^***^ | 0.76^***^ (0.42, 1.09) | 0.17 | 0.76^***^ (0.43, 1.10) | 0.17 | –0.01 (–0.02, 0.01) | 0.01 | NA |
|  |  | ≥65 | 0.27 | 35.21^***^ | 0.56 (–0.02, 1.13) | 0.29 | 0.57 (0.00, 1.15) | 0.29 | –0.02 (–0.06, 0.01) | 0.02 | NA |
|  | Exposure to violence | <65 | 0.31 | 93.21^***^ | 1.06^***^ (0.69, 1.43) | 0.19 | 1.08^***^ (0.71, 1.45) | 0.19 | –0.02 (–0.05, –0.004) | 0.01 | 2.25% |
|  |  | ≥65 | 0.31 | 30.04^***^ | 1.10^**^ (0.44, 1.77) | 0.34 | 1.13^***^ (0.46, 1.79) | 0.34 | –0.02 (–0.10, 0.04) | 0.03 | NA |
|  | Parent/sibling death or disability | <65 | 0.29 | 100.09^***^ | 0.73^***^ (0.38, 1.07) | 0.18 | 0.73^***^ (0.38, 1.08) | 0.18 | –0.01 (–0.03, 0.01) | 0.01 | NA |
|  |  | ≥65 | 0.28 | 46.50^***^ | 0.49 (–0.07, 1.04) | 0.28 | 0.50 (–0.05, 1.05) | 0.28 | –0.01 (–0.06, 0.03) | 0.02 | NA |
|  | Parental maladjustment | <65 | 0.34 | 70.57^***^ | 1.86^***^ (1.36, 2.37) | 0.26 | 1.90^***^ (1.39, 2.40) | 0.26 | –0.03 (–0.07, –0.001) | 0.02 | 1.62% |
|  |  | ≥65 | 0.34 | 28.74^***^ | 2.03^***^ (1.25, 2.81) | 0.40 | 2.03^***^ (1.25, 2.81) | 0.40 | –0.01 (–0.05, 0.02) | 0.02 | NA |

Estimated by the bias-corrected percentile bootstrap method.

ACEs, adverse childhood experiences. DS, depressive symptoms. B, effect estimate coefficient. LLCI, lower limits of 95% confidence interval. ULCI, upper limits of 95% confidence interval. SE, standard error. Mediation proportion = | Indirect effect | / Total effect. NA indicates that the mediation effect for the corresponding path was not significant and thus not calculated.

^*^ *p* < 0.05, ^**^ *p* < 0.01, ^***^ *p* < 0.001.

**Table S6** Balance test for post-matching discovery dataset

| Variable | Matching situation |  | Child maltreatment | Exposure to violence | Parent/sibling death or disability | Parental maladjustment | Controls | *p* value | *SMD* |
| --- | --- | --- | --- | --- | --- | --- | --- | --- | --- |
| Age | Pre-matching |  | 60.00  (53.00-66.00) | 58.00  (52.00-66.00) | 62.00  (54.00-68.00) | 62.00  (54.00-68.00) | 59.00  (53.00-66.00) | < 0.001^a^ | 0.14 |
|  | Post-matching |  | 62.00  (54.00-68.00) | 62.00  (54.00-68.00) | 62.00  (54.00-68.00) | 62.00  (54.00-68.00) | 62.00  (54.00-68.00) | 0.93^a^ | 0.01 |
| Male or Female Birth Gender | Pre-matching | Female | 1,975 (45.00%) | 1,360 (49.51%) | 2,274 (49.97%) | 726 (54.06%) | 1,426 (54.87%) | < 0.001^b^ | 0.10 |
|  |  | Male | 2,414 (55.00%) | 1,387 (50.49%) | 2,277 (50.03%) | 617 (45.94%) | 1,173 (45.13%) |  |  |
|  | Post-matching | Female | 1,405 (53.67%) | 721 (54.17%) | 2,158 (54.25%) | 726 (54.55%) | 721 (54.17%) | 0.99^b^ | 0.01 |
|  |  | Male | 1,213 (46.33%) | 610 (45.83%) | 1,820 (45.75%) | 605 (45.45%) | 610 (45.83%) |  |  |

*SMD*, standardized mean differences. ^a^ Kruskal-Wallis rank sum test, ^b^ Chi-squared test.

**Table S7** Participant characteristics of ACEs categories in the post-matching discovery dataset

| Variable | Child maltreatment  (*n* = 2,618) | Exposure to violence  (*n* = 1,331) | Parent/sibling death or disability  (*n* = 3,978) | Parental maladjustment  (*n* = 1,331) | Controls  (*n* = 1,331) | *p* value |
| --- | --- | --- | --- | --- | --- | --- |
| CES-D scores |  |  |  |  |  | < 0.001^a^ |
| Median [IQR] | 7.0 [3.0;13.0] | 8.0 [4.0;14.0] | 8.0 [4.0;13.0] | 11.0 [5.0;17.0] | 6.0 [3.0;11.0] |  |
| Social activity index |  |  |  |  |  | 0.02^a^ |
| Median [IQR] | 1.0 [0;3.0] | 1.0 [0;3.0] | 1.0 [0;3.0] | 1.0 [0;3.0] | 1.0 [0;3.0] |  |
| Age (years) |  |  |  |  |  | 0.94^b^ |
| < 65 | 1,630(62.26%) | 820(61.61%) | 2,434(61.19%) | 819(61.53%) | 819(61.53%) |  |
| ≥ 65 | 988(37.74%) | 511(38.39%) | 1,544(38.81%) | 512(38.47%) | 512(38.47%) |  |
| Male or Female Birth Gender |  |  |  |  |  | 0.99^b^ |
| Female | 1,405(53.67%) | 721(54.17%) | 2,158(54.25%) | 726(54.55%) | 721(54.17%) |  |
| Male | 1,213(46.33%) | 610(45.83%) | 1,820(45.75%) | 605(45.45%) | 610(45.83%) |  |
| Education level |  |  |  |  |  | < 0.001^b^ |
| < Middle school | 1,526(58.29%) | 846(63.56%) | 2,612(65.66%) | 999(75.06%) | 788(59.20%) |  |
| ≥ Middle school | 1,092(41.71%) | 485(36.44%) | 1,366(34.34%) | 332(24.94%) | 543(40.80%) |  |
| Marital status |  |  |  |  |  | 0.14^b^ |
| No spouse | 322(12.30%) | 173(13.00%) | 482(12.12%) | 191(14.35%) | 150(11.27%) |  |
| Have a spouse | 2,296(87.70%) | 1,158(87.00%) | 3,496(87.88%) | 1,140(85.65%) | 1,181(88.73%) |  |
| Chronic diseases |  |  |  |  |  | < 0.001^b^ |
| No | 519(19.82%) | 231(17.36%) | 685(17.22%) | 205(15.40%) | 305(22.92%) |  |
| Yes | 2,099(80.18%) | 1,100(82.64%) | 3,293(82.78%) | 1,126(84.60%) | 1,026(77.08%) |  |
| BADL disability |  |  |  |  |  | < 0.001^b^ |
| No | 2,210(84.42%) | 1,077(80.92%) | 3,255(81.83%) | 1,015(76.26%) | 1,135(85.27%) |  |
| Yes | 408(15.58%) | 254(19.08%) | 723(18.17%) | 316(23.74%) | 196(14.73%) |  |
| IADL disability |  |  |  |  |  | :< 0.001^b^ |
| No | 2,102(80.29%) | 1,009(75.81%) | 3,068(77.12%) | 923(69.35%) | 1,088(81.74%) |  |
| Yes | 516(19.71%) | 322(24.19%) | 910(22.88%) | 408(30.65%) | 243(18.26%) |  |
| Self-rated health status |  |  |  |  |  | < 0.001^b^ |
| Unhealthy | 1,968(75.17%) | 1,043(78.36%) | 3,090(77.68%) | 1,060(79.64%) | 949(71.30%) |  |
| Healthy | 650(24.83%) | 288(21.64%) | 888(22.32%) | 271(20.36%) | 382(28.70%) |  |
| Childhood health history |  |  |  |  |  | < 0.001^b^ |
| Unhealthy | 348(13.29%) | 234(17.58%) | 622(15.64%) | 262(19.68%) | 97(7.29%) |  |
| Healthy | 2,270(86.71%) | 1,097(82.42%) | 3,356(84.36%) | 1,069(80.32%) | 1,234(92.71%) |  |
| Smoking |  |  |  |  |  | 0.87^b^ |
| No | 1,947(74.37%) | 988(74.23%) | 2,922(73.45%) | 980(73.63%) | 972(73.03%) |  |
| Yes | 671(25.63%) | 343(25.77%) | 1,056(26.55%) | 351(26.37%) | 359(26.97%) |  |
| Drinking |  |  |  |  |  | 0.02^b^ |
| No | 1,646(62.87%) | 845(63.49%) | 2,593(65.18%) | 895(67.24%) | 891(66.94%) |  |
| Yes | 972(37.13%) | 486(36.51%) | 1,385(34.82%) | 436(32.76%) | 440(33.06%) |  |
| Sleep duration |  |  |  |  |  | < 0.001^b^ |
| Abnormal | 1,064(40.64%) | 617(46.36%) | 1,720(43.24%) | 656(49.29%) | 499(37.49%) |  |
| Normal | 1,554(59.36%) | 714(53.64%) | 2,258(56.76%) | 675(50.71%) | 832(62.51%) |  |
| Parent’s education level |  |  |  |  |  | 0.001^b^ |
| < Middle school | 2,303(87.97%) | 1,166(87.60%) | 3,610(90.75%) | 1,196(89.86%) | 1,158(87.00%) |  |
| ≥ Middle school | 315(12.03%) | 165(12.40%) | 368(9.25%) | 135(10.14%) | 173(13.00%) |  |
| Childhood family’s financial status |  |  |  |  |  | < 0.001^b^ |
| Worse than others | 1,058(40.41%) | 677(50.86%) | 1,793(45.07%) | 771(57.93%) | 350(26.30%) |  |
| Equal to or better than others | 1,560(59.59%) | 654(49.14%) | 2,185(54.93%) | 560(42.07%) | 981(73.70%) |  |
| Medical insurance |  |  |  |  |  | 0.22^b^ |
| No | 75(2.86%) | 35(2.63%) | 97(2.44%) | 39(2.93%) | 23(1.73%) |  |
| Yes | 2,543(97.14%) | 1,296(97.37%) | 3,881(97.56%) | 1,292(97.07%) | 1,308(98.27%) |  |
| Pension |  |  |  |  |  | 0.41^b^ |
| No | 230(8.79%) | 129(9.69%) | 405(10.18%) | 125(9.39%) | 121(9.09%) |  |
| Yes | 2,388(91.21%) | 1,202(90.31%) | 3,573(89.82%) | 1,206(90.61%) | 1,210(90.91%) |  |

ACEs, adverse childhood experiences. CES-D, the Center for Epidemiologic Studies Depression Scale. BADL, basic activities of daily living. IADL, instrumental activities of daily living. CES-D scores and social activities indexes are presented as median [interquartile range]. Other variables are presented as frequency and percentage.

^a^ Kruskal-Wallis rank sum test; ^b^ Chi-squared test.

**Table S8** Social activity as a mediator between ACEs categories and DS in the post-matching discovery dataset

| Independent variables | Model fit | | Total effect | | Direct effect | | Indirect effect | | Mediation proportion |
| --- | --- | --- | --- | --- | --- | --- | --- | --- | --- |
|  | *R*^2^ | *F* | B (LLCI, ULCI) | SE | B (LLCI, ULCI) | SE | B (LLCI, ULCI) | SE |  |
| Child maltreatment | 0.25 | 73.54^***^ | 0.87^***^ (0.50, 1.24) | 0.19 | 0.90^***^ (0.53, 1.27) | 0.19 | –0.03 (–0.06, –0.01) | 0.02 | 3.45% |
| Exposure to violence | 0.27 | 54.83^***^ | 1.32*** (0.87, 1.76) | 0.23 | 1.35^***^ (0.91, 1.79) | 0.23 | –0.03 (–0.08, –0.002) | 0.02 | 2.27% |
| Parent/sibling death or disability | 0.26 | 104.96^***^ | 0.92^***^ (0.56, 1.28) | 0.18 | 0.94^***^ (0.58, 1.31) | 0.18 | -0.03 (–0.06, 0.003) | 0.02 | NA |
| Parental maladjustment | 0.31 | 64.73^***^ | 2.40^***^ (1.91, 2.89) | 0.25 | 2.44^***^ (1.96, 2.93) | 0.25 | –0.04 (–0.10, 0.001) | 0.03 | NA |

Estimated by the bias-corrected percentile bootstrap method.

ACEs, adverse childhood experiences. DS, depressive symptoms. B, effect estimate coefficient. LLCI, lower limits of 95% confidence interval. ULCI, upper limits of 95% confidence interval. SE, standard error. Mediation proportion = | Indirect effect | / Total effect. NA indicates that the mediation effect for the corresponding path was not significant.

^*^ *p* < 0.05, ^**^ *p* < 0.01, ^***^ *p* < 0.001.

**Table S9** Multiple linear regression analysis in the replication dataset

|  | Social activity index | | CES-D scores | | CES-D scores | |
| --- | --- | --- | --- | --- | --- | --- |
|  | Model 1 | | Model 2 | | Model 3 | |
|  | B | SE | B | SE | B | SE |
| Age | 0.04 | 0.05 | –0.63^***^ | 0.13 | –0.62^***^ | 0.13 |
| Male or Female Birth Gender | –0.39^***^ | 0.05 | –1.15^***^ | 0.15 | –1.21^***^ | 0.15 |
| Education level | 0.46^***^ | 0.04 | –1.18^***^ | 0.12 | –1.11^***^ | 0.12 |
| Marital status | –0.12 | 0.07 | –1.27^***^ | 0.19 | –1.29^***^ | 0.18 |
| Chronic diseases | 0.23^***^ | 0.06 | 0.51^***^ | 0.15 | 0.54^***^ | 0.15 |
| BADL disability | –0.18^**^ | 0.06 | 2.44^***^ | 0.17 | 2.41^***^ | 0.17 |
| IADL disability | –0.14* | 0.06 | 2.97^***^ | 0.17 | 2.95^***^ | 0.18 |
| Self-rated health status | 0.23^***^ | 0.05 | –2.44^***^ | 0.14 | –2.40^***^ | 0.14 |
| Childhood health history | 0.03 | 0.06 | –0.85^***^ | 0.18 | –0.85^***^ | 0.18 |
| Smoking | 0.10 | 0.05 | 0.18 | 0.15 | 0.19 | 0.15 |
| Drinking | 0.38^***^ | 0.05 | –0.61^***^ | 0.13 | –0.55^***^ | 0.13 |
| Sleep duration | 0.02 | 0.04 | –2.12^***^ | 0.12 | –2.11^***^ | 0.12 |
| Parent’s education level | 0.34^***^ | 0.06 | –0.36^*^ | 0.17 | –0.31 | 0.17 |
| Childhood family’s financial status | 0.06^***^ | 0.05 | –0.47^***^ | 0.12 | –0.46^***^ | 0.12 |
| Medical insurance | 0.17 | 0.12 | –0.33 | 0.33 | –0.30 | 0.32 |
| Pension | 0.14* | 0.06 | 0.06 | 0.17 | 0.08 | 0.17 |
| ACEs scores | 0.04** | 0.02 | 0.44^***^ | 0.04 | 0.45^***^ | 0.04 |
| Social activity index |  |  |  |  | –0.15^***^ | 0.03 |
|  | *R*^2^ = 0.04 | | *R*^2^ = 0.28 | | *R*^2^ = 0.28 | |
|  | *F*_(17, 8881)_ = 21.3^***^ | | *F*_(17, 8881)_ = 203.5^***^ | | *F*_(18, 8880)_ = 194.2^***^ | |

ACEs, adverse childhood experiences. CES-D, the Center for Epidemiologic Studies Depression Scale. BADL, basic activities of daily living. IADL, instrumental activities of daily living.

Model 1 examined the link between ACEs and social activity. Model 2 explored the relationship between ACEs and DS. Model 3 assessed whether social activity mediated the relationship between ACEs and DS. B, regression coefficient. SE, standard error.

^*^ *p* < 0.05, ^**^ *p* < 0.01, ^***^ *p* < 0.001.

**Table S10** Social activity as a mediator between ACEs categories and DS in the replication dataset

| Independent variables | Model fit | | Total effect | | Direct effect | | Indirect effect | | Mediation proportion |
| --- | --- | --- | --- | --- | --- | --- | --- | --- | --- |
|  | *R*^2^ | *F* | B (LLCI, ULCI) | SE | B (LLCI, ULCI) | SE | B (LLCI, ULCI) | SE |  |
| ACEs | 0.28 | 194.21^***^ | 0.44 (0.36, 0.53) | 0.04 | 0.45 (0.36, 0.53) | 0.04 | –0.01 (–0.01, –0.002) | 0.003 | 2.27% |
| Child maltreatment | 0.27 | 123.65^***^ | 0.71^***^ (0.42, 1.00) | 0.15 | 0.72^***^ (0.43, 1.01) | 0.15 | –0.01 (–0.03, 0.004) | 0.01 | NA |
| Exposure to violence | 0.32 | 120.71^***^ | 1.05^***^ (0.73, 1.38) | 0.17 | 1.08^***^ (0.76, 1.40) | 0.16 | –0.03 (–0.05, –0.01) | 0.01 | 2.86% |
| Parent/sibling death or disability | 0.29 | 139.91^***^ | 0.67^***^ (0.38, 0.96) | 0.15 | 0.68^***^ (0.38, 0.97) | 0.15 | –0.01 (–0.03, 0.01) | 0.01 | NA |
| Parental maladjustment | 0.34 | 94.30^***^ | 1.92^***^ (1.50, 2.34) | 0.22 | 1.94^***^ (1.52, 2.37) | 0.22 | –0.02 (–0.05, –0.002) | 0.01 | 1.04% |

Estimated by the bias-corrected percentile bootstrap method.

ACEs, adverse childhood experiences. DS, depressive symptoms. B, effect estimate coefficient. LLCI, lower limits of 95% confidence interval. ULCI, upper limits of 95% confidence interval. SE, standard error. Mediation proportion = | Indirect effect | / Total effect. NA indicates that the mediation effect for the corresponding path was not significant and thus not calculated.

^*^ *p* < 0.05, ^**^ *p* < 0.01, ^***^ *p* < 0.001.

**References**

1. Wang YL. *A study on the social activities of the elderly in China and their influence*. Changchun: Jilin University; 2023. Doctoral dissertation.

2. Yang F, Su Q, Ran Q, Hu YY. Longitudinal change of intrinsic capacity and associated factors in older Chinese adults: China Health and Retirement Longitudinal Study. *J Nutr Health Aging*. 2024;28(5):100214. doi:10.1016/j.jnha.2024.100214.

3. Yang X, Wang YL. Quantitative analysis: Influence of social activities on the elderly health. *Popul J*. 2020;42(3):66–77. doi:10.16405/j.cnki.1004-129X.2020.03.006.

4. Zhang TT, Kan LN, Jin CB, Shi WM. Adverse childhood experiences and their impacts on subsequent depression and cognitive impairment in Chinese adults: A nationwide multi-center study. *J Affect Disord* 2023;323:884–892. doi:10.1016/j.jad.2022.12.058.

5. Ho DE, Imai K, King G, Stuart EA. MatchIt: Nonparametric preprocessing for parametric causal inference. *J Stat Softw*. 2011;42(8). doi:10.18637/jss.v042.i08.

6. Lin L, Wang HHX, Lu CY, Chen WQ, Guo VY. Adverse childhood experiences and subsequent chronic diseases among middle-aged or older adults in China and associations with demographic and socioeconomic characteristics. *JAMA Netw Open*. 2021;4(10):e2130143. doi:10.1001/jamanetworkopen.2021.30143.
